# Nano zinc supplementation in broiler nutrition: current evidence, mechanisms of action, and future perspectives

**DOI:** 10.3389/fvets.2026.1925519

**Published:** 2026-09-17

**Authors:** Desiderio Rodriguez Velazquez, Babak Hosseintabar-Ghasemabad, Jorge Antonio Varela Guerrero, Ivan F. Gorlov, Marina I. Slozhenkina, Aleksandr A. Mosolov, Silvia Ondrašovičová, Alireza Seidavi, José Eduardo García Martínez, Francisco Alonso Rodríguez Huerta, Susanne Kreuzer-Redmer, Mona M. M. Y. Eghandour

**Affiliations:** 1 Facultad de Medicina Veterinaria y Zootecnia, Universidad Autónoma del Estado de México, Toluca, Mexico; 2 Department of Animal Science, Faculty of Agriculture, University of Tabriz, Tabriz, Iran; 3 Volga Region Research Institute of Manufacture and Processing of Meat and Milk Production, Volgograd, Russia; 4 Department of Biology and Physiology, University of Veterinary Medicine and Pharmacy in Košice, Košice, Slovakia; 5 Department of Animal Science, Ra.C., Islamic Azad University, Rasht, Iran; 6 Department of Animal Nutrition, Antonio Narro Autonomous Agrarian University, Saltillo, Coahuila, Mexico; 7 Centre for Animal Nutrition and Welfare, Clinical Department for Farm Animals and Food System Transformation, University of Veterinary Medicine Vienna, Vienna, Austria

**Keywords:** bioavailability, broiler chickens, immune function, intestinal health, oxidative stress, sustainability, zinc nanoparticles

## Abstract

Zinc is an essential trace mineral involved in numerous biological processes, including growth, antioxidant defense, immune function, and intestinal integrity in broiler chickens. Optimizing its bioavailability remains an important objective in modern poultry nutrition. Nanotechnology has emerged as a promising strategy to enhance zinc bioavailability while potentially reducing dietary inclusion levels and environmental zinc excretion. Current evidence suggests that nano zinc supplementation may enhance antioxidant capacity, modulate immune responses, support intestinal health, and improve productive performance under specific experimental conditions. Several studies further indicate reduced zinc excretion compared with conventional zinc sources, suggesting potential environmental benefits through lower mineral losses. Furthermore, nano zinc has been proposed as part of nutritional strategies aiming to reduce reliance on antimicrobial use by improving gut health and host resilience. However, direct evidence supporting nano zinc as an alternative to antibiotics under commercial production conditions remains limited. Nano zinc supplementation has also been associated with improvements in intestinal morphology, microbial composition, and barrier function, although the underlying molecular mechanisms remain to be fully elucidated. Despite these promising findings, important knowledge gaps remain regarding optimal particle characteristics, dosage, long-term safety, tissue accumulation, and regulatory acceptance. This review summarizes the current evidence on the biological functions and applications of nano zinc in broiler nutrition, discusses the proposed mechanisms of action, critically evaluates the available literature, and highlights priorities for future research.

## Introduction

The poultry industry faces increasing pressure to improve production efficiency while reducing environmental impact and maintaining animal health. Nutritional interventions, including functional feed additives, have received increasing attention in both human and animal nutrition because of their potential to improve health, physiological functions, and production efficiency (1–9). Among emerging nutritional approaches, nanotechnology has attracted considerable interest because of its potential to enhance nutrient bioavailability, improve feed efficiency, and reduce nutrient losses to the environment (10–12). Consequently, optimizing the bioavailability and utilization of essential trace minerals, particularly zinc, has become an important objective in modern broiler nutrition. Recent research has highlighted nanotechnology as a promising strategy to improve the bioavailability of trace minerals while potentially reducing mineral excretion and environmental contamination (13). Although the application of nanotechnology in poultry nutrition is still evolving, zinc nanoparticles have attracted particular attention because of their favorable physicochemical properties and their potential to enhance zinc utilization at lower dietary inclusion levels. Nevertheless, the optimal particle characteristics, supplementation strategies, and long-term safety of nano zinc require further investigation before broad practical implementation. In addition to improving zinc bioavailability, nano zinc supplementation has been investigated as a potential strategy to support productive performance, antioxidant defense, immune function, intestinal health, and environmental sustainability. However, the reported responses vary considerably among studies, emphasizing the need for a critical evaluation of the available evidence and remaining knowledge gaps (13–17).

## Methodological approach

The importance of nanotechnology in the poultry industry, followed by the importance of zinc in poultry, was discussed, with a focus on the research of nano zinc in broiler chickens. Articles from the past decade were searched using Google Scholar, ScienceDirect, Scopus, Web of Science, and PubMed databases with the keywords of nanotechnology, zinc, nano zinc oxide (Nano-ZnO), broilers, and poultry nutrition. Particular emphasis was placed on studies evaluating the effects of nano zinc on growth performance, antioxidant status, immune function, intestinal physiology, intestinal microbiota, nutrient utilization, environmental sustainability, and food safety.

In order to enhance the visual quality of the article, the design figures and schematic diagrams were graphically enhanced using artificial intelligence-based tools. The use of these tools was limited solely to improving the quality of presentation, layout, and graphical display of the figures and had no role in the generation of scientific data, analysis of information, interpretation of results, or conclusions of the article. All scientific concepts, content of the figures, and their final version were prepared, reviewed, and approved by the authors.

## Application and importance of nanotechnology in poultry

Nanotechnology refers to the manipulation and application of materials with dimensions between approximately 1 and 100 nm, where unique physicochemical properties emerge compared with their bulk counterparts (18, 19). Owing to their increased surface area, altered surface reactivity, and improved cellular interactions, nanoparticles have attracted considerable interest across medicine, agriculture, and animal nutrition as innovative tools to improve the delivery and biological availability of nutrients and bioactive compounds (18–20).

In poultry nutrition, nanotechnology has been proposed as a promising approach to improve nutrient utilization while reducing environmental losses. The enhanced bioavailability of nano-sized compounds may allow lower dietary inclusion rates without compromising biological efficacy. Consequently, nanotechnology has been identified as one of the emerging technologies with considerable potential for future feed additive development (19–22). Among nutritional applications, trace minerals represent particularly suitable candidates for nano formulation because their bioavailability is often limited by physicochemical interactions within the gastrointestinal tract. Reducing particle size increases the surface-to-volume ratio, thereby potentially enhancing mineral solubility, intestinal absorption, and biological utilization while simultaneously decreasing mineral excretion into the environment (21, 23, 24). Beyond improved bioavailability, nanoparticles have been associated with enhanced nutrient delivery, modulation of oxidative status, antimicrobial activity, immune regulation, and improved nutrient digestibility. Several experimental studies further suggest beneficial effects on growth performance, feed efficiency, and intestinal function in poultry, although responses vary depending on particle characteristics, dosage, and experimental conditions (10, 17, 24–33).

Optimizing nutrient utilization through innovative feed additives may contribute not only to improved animal performance but also to enhanced product quality and sustainability. In this context, nanotechnology represents one of several promising approaches currently under investigation (34–36).

The biological behavior of nanoparticles is strongly influenced by particle size, surface properties, and physicochemical characteristics. Smaller particles generally exhibit enhanced tissue penetration and cellular interactions, which may increase biological efficacy but also raise important questions regarding biodistribution, persistence, and long-term safety (25, 26, 37–40).

Although numerous experimental studies have reported promising effects of nano feed additives, their practical application requires careful evaluation of efficacy, safety, manufacturing consistency, regulatory approval, and consumer acceptance (21, 22, 41). Besides their nutritional potential, the application of nanotechnology in animal production is accompanied by scientific, ethical, and regulatory considerations (Figure 1).

**Figure 1:**
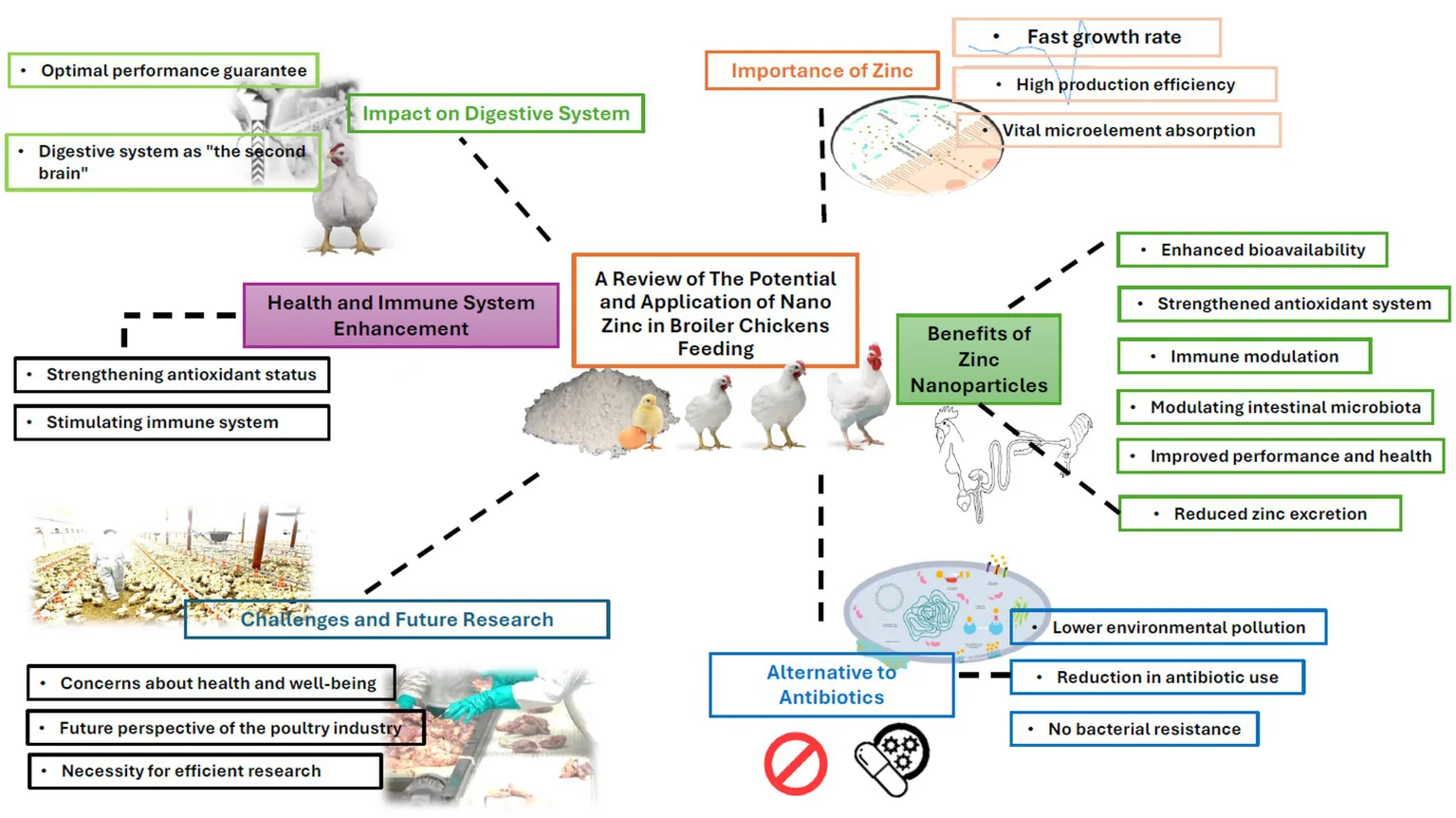
Objectives of nanotechnology for feed additives.

The application of nanotechnology in animal nutrition has also raised important questions regarding safety, environmental impact, animal welfare, and consumer acceptance (42). Potential concerns include tissue accumulation, unintended biological interactions, nanoparticle persistence, and regulatory challenges associated with their use in food-producing animals. Although current evidence indicates considerable potential for nano-based feed additives, further research is required to establish standardized production methods, evaluate long-term safety, and support evidence-based regulatory frameworks (42–45).

## Importance of zinc in poultry

Zinc is an essential trace element required for numerous physiological processes in poultry, including growth, skeletal development, antioxidant defense, immune competence, epithelial integrity, and reproductive performance (16, 20, 46). Owing to its role as a structural, catalytic, and regulatory component of more than 300 enzymes and numerous zinc-finger proteins, adequate zinc supply is indispensable for normal cellular metabolism and gene regulation (20, 46, 47).

Commercial poultry diets are routinely supplemented with zinc to meet physiological requirements. However, the bioavailability of conventional inorganic zinc sources is often limited by interactions with dietary components such as phytate, calcium, and other minerals, resulting in relatively low absorption efficiency and considerable zinc excretion (16, 48–50). Consequently, strategies that improve zinc bioavailability have become increasingly important from both nutritional and environmental perspectives.

Currently, zinc is supplemented predominantly as inorganic (e.g., zinc oxide or zinc sulfate) or organic zinc complexes. Although organic sources generally exhibit higher bioavailability than inorganic salts, they are associated with higher production costs, whereas inorganic sources remain the most widely used because of their availability and economic feasibility (48, 49). Nano zinc has emerged as a potential alternative that may combine improved bioavailability with lower dietary inclusion levels. Because zinc acts as a structural, catalytic, and regulatory cofactor for more than 300 enzymes and numerous zinc-finger proteins, it participates in a wide range of physiological processes. Representative zinc-dependent enzymes and their major biological functions are summarized in Table 1.

**Table 1:** List of zinc metalloenzymes in six enzyme classes.

Biological function	The most important zinc-dependent proteins and enzymes	Related molecular pathways and cellular targets	Physiological significance in poultry	Evidence related to nano zinc
Antioxidant defense	Cu/Zn-dependent superoxide dismutase (SOD1)	Activation of Nrf2/ARE pathway, increased expression of antioxidant enzymes, decreased ROS production, induction of metallothionein	Protection against oxidative stress, decreased lipid peroxidation, maintained cellular health	There is extensive direct evidence; nano zinc has increased SOD, GPx, and TAC activity and decreased MDA.
Immune system regulation	Thymolin, Zinc-finger transcription factors	Inhibition of the NF-κB pathway, reduction of TNF-*α*, IL-1β and IL-6 expression, regulation of T lymphocyte and macrophage activity	Maturation of T lymphocytes, regulation of inflammatory responses and enhancement of immunity	There is extensive direct evidence; nano zinc modulates the immune response and cytokine expression.
Maintain gut health and integrity	Alkaline phosphatase, tight junction proteins (Occluding, Claudin, ZO-1)	Regulate tight junction proteins, reduce mucosal inflammation, enhance epithelial barrier function, maintain microbiota balance	Increase epithelial health, reduce intestinal permeability, and improve nutrient absorption	There is direct evidence in nano zinc studies; improvement in gut morphology and mucosal barrier function has been reported.
Growth and protein synthesis	RNA polymerase, DNA polymerase	Activation of zinc-dependent enzymes, increased DNA and RNA synthesis, stimulation of cell proliferation	Rapid growth, cell proliferation and increased production performance	Mostly based on the general physiology of zinc; nano zinc enhances these processes due to its higher bioavailability.
Gene expression regulation	Zinc-finger proteins	Regulate the binding of transcription factors to DNA, regulate gene expression, interact with cell signaling pathways	Control gene transcription and regulate metabolic and immune functions	Mainly based on zinc physiology; nano zinc studies have also reported altered expression of some functional genes.
Bone growth and development	Carbonic anhydrase, alkaline phosphatase	Regulation of osteoblast differentiation, bone mineralization, regulation of metalloenzyme activity	Skeletal growth, bone strength, and proper mineralization	Direct evidence is limited; observed effects are likely due to increased zinc bioavailability.
Energy metabolism	Alcohol dehydrogenase, lactate dehydrogenase	Regulate metalloenzyme activity, maintain mitochondrial function, improve energy metabolism	Increase metabolic efficiency and nutrient utilization	Mainly based on zinc physiology; indirect evidence exists in nano zinc studies.

SOD, Superoxide Dismutase; Nrf2, Nuclear Factor Erythroid 2-Related Factor 2; ARE, Antioxidant Response Element; GPx, Glutathione Peroxidase; TAC, Total Antioxidant Capacity; MDA, Malondialdehyde; NF-κB, Nuclear Factor Kappa B; TNF-α, Tumor Necrosis Factor-Alpha; IL-1, Interleukin-1; RNA, Ribonucleic Acid; DNA, Deoxyribonucleic Acid.

One of the best-characterized biological functions of zinc is its role as a cofactor for numerous metalloenzymes involved in acid–base regulation, antioxidant defense, and cellular metabolism. Furthermore, zinc-binding proteins such as metallothioneins contribute to intracellular zinc homeostasis, protection against oxidative stress, and heavy metal detoxification (50–52). Figure 2 summarizes the principal biological functions of zinc in broiler chickens, including its involvement in antioxidant defense, immune regulation, intestinal health, growth, and productive performance. These diverse physiological functions provide the biological basis for the increasing interest in improving zinc bioavailability through nano formulations.

**Figure 2:**
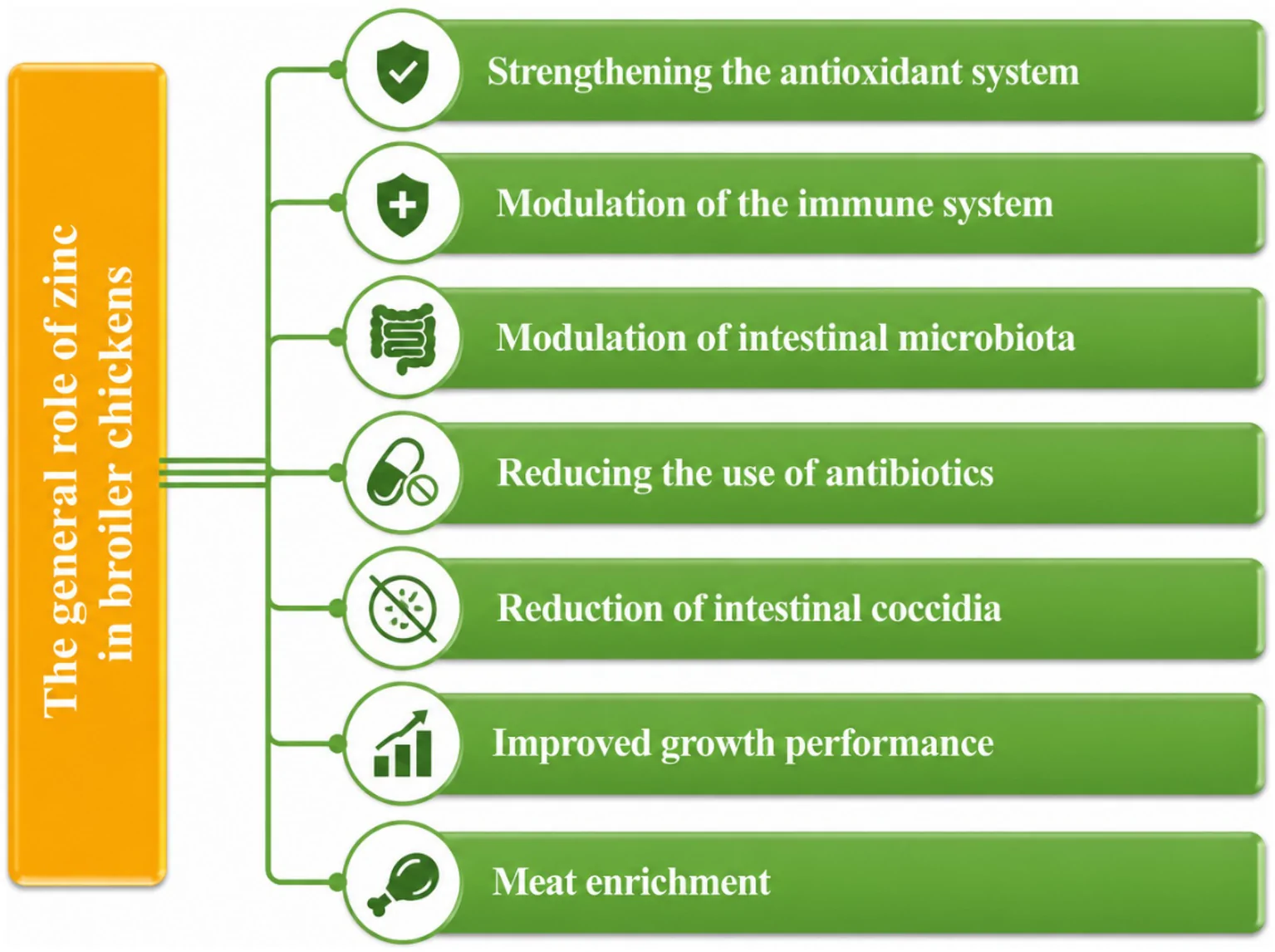
The general role of zinc in broiler chickens.

Another vital role of zinc is to help regulate the immune system and ensure health in the form of strengthening the antioxidant defense system in broiler chickens. However, one of the goals of poultry farming is to prevent the occurrence of diseases through the correct implementation of biosecurity programs. By being present in the structural compounds of various proteins and independent enzymes such as superoxide dismutase, zinc can strengthen and support the activity of this enzyme. This enzyme is an essential enzyme in the antioxidant defense system and as a metalloenzyme exists in most of the metabolic pathways of aerobic organisms and plays a key role in protecting organisms against the toxic effects of reactive oxygen species, especially against radicals (53). Superoxide dismutase catalyzes the dismutation reaction and converts superoxide radicals into oxygen and hydrogen peroxide molecules. Superoxide dismutase has three types of isozymes with metal cofactors. Copper-Zinc superoxide dismutase is widely distributed in eukaryotes. While the enzymes with manganese and iron are mainly found in the mitochondria of prokaryotes. Each molecule of eukaryotic copper-zinc superoxide is composed of approximately 153 amino acids with dimers of identical subunits. In addition to the role of superoxide dismutase in protecting cells against superoxide radicals, studies have shown that zinc can prevent lipid peroxidation by iron by blocking iron binding sites on the cell surface with the help of vitamin E (20, 54).

Zinc deficiency has led to increased susceptibility to infectious diseases in poultry, which indicates the importance of zinc in the immune system. Zinc deficiency can lead to a decrease in the defense mechanism of the host body in different ways, which may be different from the skin of the first defense barrier to humoral and cellular immunity (20, 55). The zinc element has an effective and positive role on the normal functioning of the system by increasing the number of thymocytes (lymphocytes derived from the thymus gland) and T cells, as well as the production of interferon and interleukin. In addition, zinc is a necessary cofactor for the hormone thymolin. Thymolin is a thymus hormone that binds to receptors on the surface of T lymphocytes and causes the maturation and activity of T lymphocytes. Zinc is connected to thymolin through asparagine side chains and hydroxyl groups and forms the active form of thymolin (16, 56). Intestinal absorption of zinc takes place through active transport or facilitated diffusion, and in broilers, the ileum is the main site of zinc absorption (57). Zinc absorption does not depend on age, but with some nutritional factors such as some amino acids, lactose and the presence of low amounts of iron in the diet, it has a negative effect on its absorption. Deficiency of proteins and increase in dietary phytate reduces the absorption of zinc in the body. Increasing calcium absorption can aggravate this deficiency (50).

In addition, activators such as picolinic acid secreted by the pancreas, vitamin B6 (which increases the amount of picolinic acid), citrate, and amino acids such as histidine, lysine, cysteine, and methionine play an active role during the zinc absorption process. After absorption, zinc is transferred to the liver and binds with albumin. The main route of zinc excretion is feces and then through the kidneys and skin (20, 50, 56).

Considering the importance of helping the environment and the place of metals in nature, reducing their disposal in the environment is a fundamental issue and aids in reducing the pressure on nature. In the normal commercial diet, 94% of zinc enters the feces. High amounts of zinc consumed lead to less use of this element and subsequently decrease zinc retention (6%) in the body (58).

Zinc disposal with diets containing low amounts of zinc or using zinc sources with high bioavailability is considered a helpful strategy in the poultry industry in order to serve the environment.

In general, in a series of research studies, the role of zinc in strengthening the integrity of the intestine and helping to reduce the expression of pro-inflammatory cytokines and adjusting the intestinal microbiota has been confirmed, and promising results have been recorded (59). Coccidiosis is known as an intestinal disease caused by *Eimeria* protozoa that can affect the health of broiler chickens (59, 60). It is known that the proliferation of *Eimeria* in the intestinal epithelium leads to severe tissue damage in the form of destruction of enterocytes and affects the welfare and health of the flock (59, 60). The fatal effects of reduced productivity caused by coccidiosis in the poultry industry create an annual medical burden of more than two billion dollars (59, 61, 62).

When chickens are exposed to coccidiosis, the structure of the intestinal microbiota is changed and the microbial diversity in the cecum is greatly reduced. The reduction of members of the *Ruminococcaceae* family is the largest, and in parallel, the production and content of short-chain fatty acids (SCFA), which are the energy source of beneficial bacteria, are greatly reduced in the cecum (59, 63, 64). As one of the most important microelements, zinc is effective in modulating endogenous defense mechanisms against infections, inflammations and oxidative stress, which will be doubled if absorption and bioavailability are enhanced. Minerals in the upper parts of the digestive tract (duodenum and distal intestine) tend to separate and are better absorbed due to the low pH. In the lower parts of the digestive tract, there is a possibility of interaction between mineral cations and other components of the material, leading to the formation of insoluble complexes with low accessibility (59, 65, 66).

The most well-known insoluble complex in the diet of broiler chickens is phytate, in which cations of macro and microelements, especially calcium, zinc, and copper, are connected to phytic acid by increasing the pH above 4, and the insoluble complex is formed. Because of this, the availability of minerals and the performance of phytases decreased (16, 67, 68). Zinc is known as a micronutrient with effective performance in healing the damage caused by intestinal diseases of chickens (69). These authors contend that the necessity of consuming zinc resources in feeding broilers is always higher than the recommended concentration in the standard tables (16, 70). Zinc nutrition is mainly in broilers for many biological and metabolic functions, including the immune response against the challenge of pathogens, growth and quality meat is essential (16, 71).

When challenged by disease and infections, the distribution of zinc in the animal’s body is changed, and the levels of consumption probably increase (72). The effect of zinc on growth performance and antioxidant system (16, 66). Strengthening immune defense and inflammation, helping intestinal permeability, and modulating intestinal microbiota are very important in many research studies (16). Zinc can increase the number of intestinal enterobacteria (73), and also restore the cecal microflora of chickens infected with *Salmonella typhimurium* by increasing beneficial *Lactobacillus* bacteria and reducing *Salmonella colonization* (74). Bortoluzzi et al. (16) in their review report noted the concern about the increase of intestinal infections in broilers due to the reduction in use of antibiotics and ionophores for suppression or prevention of coccidia in the diet of trending broiler chickens. Issues such as bacterial resistance, rock erosion, and environmental pollution due to the consumption of high doses of some minerals in poultry nutrition have intensified this concern. Therefore, maximizing the absorption of minerals in the digestive tract is a practical proposal in the future perspective of poultry feed additives in order to replace antibiotics and adjust the immune system (14, 19). A review of scientific literature shows that zinc can play an effective role in the growth and regeneration of the intestinal tissue in chickens and is a reliable solution to overcome the challenges of *Coccidia* and *Clostridium perfringens* (16).

Another use of zinc in poultry, especially in broiler chickens, is to enrich products and to improve the consumer’s health. However, one of the goals of nutritional science is to increase nutritional value without compromising its quality. Meat products are one of the important sources of zinc, iron, copper, and selenium, which have a high biological capacity for humans. In many societies, in order to meet the needs of the body, the use of natural food sources, including meat, is a reliable solution. The department always has many fans. Therefore, meat products enriched with some elements such as zinc can be very important in improving the health of society (75). Scientific reviews and surveys in developed countries have shown that food fortification is the best and most reliable method to eliminate nutritional deficiencies. Therefore, the enrichment of zinc element in poultry meat can play a significant role in meeting the needs of consumers and, on the other hand, solve the deficiency of this element (20). A review of the scientific literature review shows that supplementing the diet with zinc increases the concentration of zinc in the bones, liver, kidneys and muscles of broiler chickens. Researchers have shown that the retention of zinc in the meat and different components of the carcass after zinc supplementation demonstrates an increasing trend post-treatment (76). In addition, when the amount of zinc in the diet is higher than the growth requirements of chickens, the concentration of zinc in the plasma and tibia increases (58). In a study, it was reported that adding nano zinc oxide at concentrations 25% higher than required resulted in the best production composition of nano zinc oxide, which provided the best production performance, bone quality, and intestinal microbiota (*Lactobacillus, Enterobacteriaceae*, and *Salmonella*), and subsequently ensured herd health (77).

Collectively, the broad range of biological functions of zinc highlights the importance of ensuring an adequate and biologically available zinc supply in broiler chickens. Consequently, nutritional strategies that improve zinc utilization efficiency, such as nano zinc supplementation, have attracted considerable research interest in recent years. The following section summarizes the current evidence regarding the biological effects of nano zinc supplementation in broiler nutrition.

## Effects of nano zinc consumption in poultry nutrition

Evidence published in recent years suggests that zinc oxide nanoparticles (Nano-ZnO) can improve growth performance, gut health, immune response, and antioxidant capacity of broiler chickens through a complex set of mechanisms. However, the magnitude of these responses is not uniform across studies and depends on the physicochemical properties of the nanoparticles, synthesis method, dosage, basal diet composition, age of the birds, flock health status, and experimental conditions. Therefore, evaluating the effects of nano-zinc requires simultaneous investigation of biological mechanisms and factors affecting the variability of responses across studies (33, 50, 78, 79). In a study by Abd El-Hack et al. (79), it was reported that dietary supplementation with 0.2 and 0.4 mg/kg nano zinc improved weight gain and carcass characteristics while reducing serum cholesterol, low-density lipoprotein (LDL), and alanine aminotransferase (ALT) concentrations. Furthermore, meat composition was improved, with the 0.4 mg/kg supplementation level showing the most favorable overall response.

In addition, nano zinc exhibited antimicrobial activity against several pathogenic microorganisms, suggesting its potential as a component of nutritional strategies aimed at improving gut health and reducing the reliance on antimicrobial compounds. These beneficial effects have been attributed primarily to the small particle size and increased surface area of nanoparticles, which enhance biological interactions, improve zinc bioavailability, and may support intestinal barrier function, antioxidant capacity, and immune responses (78, 79).

El-Katcha et al. (50) hypothesized that nano zinc can play a significant role in improving flock performance by increasing the expression of genes related to metabolism and growth hormone, and insulin in broiler chickens. However, strengthening and modulating the immune system of chickens was also attributed to the increase in the production of interferon and peripheral cells and thymocytes after nano zinc feeding (79).

Arun Bharathi et al. (80) synthesized nano zinc with the ball milling technique and used this source as an additive to replace other sources of zinc in poultry feed. These researchers analyzed the synthesized product in terms of particle size and the performance of nanoparticles in terms of size and distribution, respectively, with the methods of zeta potential, X-Ray Diffraction (XRD), Fourier Transform Infra-Red spectroscopy (FTIR) and Transmission Electron Microscope (TEM). The results showed that XRD and TEM in the nanostructures of zinc oxide nanoparticles can have the most optimal performance in poultry nutrition, because zinc oxide nanoparticles synthesized using the ball mill method could achieve all the necessary characteristics of nanoparticles. and be a reliable substitute for the mineral and organic form of zinc oxide in poultry feed, and introduce a valuable source in nutrition due to its good potential in high bioavailability and low harmful environmental effects. Because zinc is supplemented in feed in the form of inorganic (zinc sulfate, zinc oxide, etc.), organic (zinc methionine, zinc protein, etc.), and nano zinc, the correct understanding and knowledge of each source in different conditions is important. Investigations show that the sulfate form is very soluble in water and allows active metal ions to increase and intensify the formation of free radicals. This then leads to the breakdown of nutrients and the destruction of fats and oils, and reduces the nutritional value of the ration. Zinc provided in the form of zinc oxide has less reactivity, but it has less bioavailability. On the other hand, organic zinc has a more favorable bioavailability, and in lower doses, it has less harmful effects on the environment without negatively affecting poultry production. However, in terms of production cost and contradictory findings, there are still challenges in using the organic form. Inorganic zinc has historically been used in poultry nutrition, but the choice is questionable for the activists of this industry with the advancement of nanotechnology and the potential of using zinc in the nano form. Use of nano technology has led to the fact that it is possible to increase the interaction of zinc with biological agents and to improve its bioavailability, and to reduce the effective dose and excretion of zinc into the environment (80). Qu et al. (81) in their research study investigated the supplementation of broiler chickens’ diets with two sizes of nano zinc. The findings showed that in the group of 82 nm and 21 nm Nano-ZnO in the amount of 40 mg/kg, the growth of immune organs, non-specific immunity, the increase of beneficial bacteria in the intestinal flora, and the ratio of the height of the villi to the depth of the crypt showed significant improvement in the small intestine of chickens compared to the control group. On the other hand, the size of nanoparticles has a negative correlation with the effects of improved traits, so that Nano-ZnO with a smaller size (21 nm) showed a stronger effect. Many studies have shown that zinc deficiency can lead to immune system atrophy, a severe reduction in the production of immune cells (81), impaired cellular immune function (82), and dysregulation of the microbiota (83) in chickens. Qu et al. (81) referred to the digestive system as the “second brain” in chickens, underscoring its importance in regulating immunity and improving flock growth and production, and in supporting microbiota adjustment. Increasing bioavailability can strengthen the efficiency of this part of the body. However, the use of nanotechnology in poultry nutrition can increase the potential of optimal use of trace elements in functional diets (84). The immunomodulatory effects of zinc lead to increased activity of macrophages, increased antibody, thymocytes and heterophil cells and can increase humoral responses (85). In addition, zinc is known as an activator of superoxide dismutase (SOD), and any improvement in bioavailability of this element is associated with an increase in antioxidant activity. However, following the strengthening of the immune system and its positive correlation with the weight gain of broiler chickens, performance improvement is not far from expected (81, 86). On the other hand, any event that leads to an increase in the size and weight of the spleen can strengthen the production and differentiation of B cells and the adaptation of the immune system (81). In a report by Zhou et al. (87), Nano-ZnO was able to promote the absorption and deposition of this element by increasing the expression of transporters in the jejunum. As a result of this event, free radicals, inflammatory mediators, and blood viscosity decreased and the ability to detoxify heavy metals in the digestive system increases (81, 88, 89). Reducing the abundance of harmful bacteria and increasing the ratio of *Firmicutes*/*Bacteroidetes* is one of the beneficial effects of using Nano-ZnO in broiler chickens, which can positively affect growth performance (81, 90). A review of scientific literature shows that *Firmicutes* and *Bacteroidetes* can play a positive and effective role in energy production and metabolism, especially starch digestion and microbial fermentation (91, 92). In research Qu et al. (81), the ratio of *Firmicutes/Bacteroidete*s was higher than 20%, which is very significant compared to the control situation, and after the consumption of Nano-ZnO, the increase in Bacteroides population had an effect on this ratio. On the other hand, it is hypothesized that *Lachnospiraceae* and *Intestinimonas* are known as butyrate producers in the intestinal microbiota, which can provide energy for intestinal epithelial cells and play an important role in inhibiting inflammation and promoting intestinal growth (93–95). Therefore, the presence of such bacteria can play a vital role in fermenting carbohydrates to produce SCFA and participating in polysaccharide metabolism, mediating colonization resistance against invading intestinal pathogens, and regulating immunity and modulating intestinal microecology (81, 95, 96).

Hatab et al. (97) conducted a research on broiler chickens by feeding 40 and 60 mg/kg of Nano-ZnO, which was produced by extracellular mycosynthesis, and the results showed that the relative weight of the spleen increased, as well as the bursa, thymus, and liver, and the relative weight loss of kidney, calculus, and intestine was significant compared to the control group. In addition, the concentration of hemoglobin, hematocrit, the number of red and white blood cells, total protein, globulin, aspartate transferase, alanine transferase, alkaline phosphatase, superoxide dismutase, glutathione peroxidase and total antioxidant capacity increased, and the concentration of malonaldehyde, uric acid and creatinine decreased significantly after consuming nano zinc sources, and all immunological organs showed tissue changes in the way of increased immunity (97). In total, biogenically synthesized Nano-ZnO supplements at the inclusion level of 40 and 60 mg/kg in the diet resulted in beneficial effects on physiological, hematological, antioxidant, and immunological indicators, and the intestinal microbial flora and health of broilers were strengthened. It was found that the concentration of 40 mg/kg in broiler diets was more efficient and a safer dose that did not have any possible harmful effects compared to higher levels.

Characteristics of the nano zinc form include smaller size, increased surface area, a high number of surface active centers, increased bioavailability, favorable absorption quality, and stronger catalytic efficiency. On the other hand, the value of this work in the case of biogenic synthesis of nanoparticles. It can be doubled, because in this type of synthesis, the use of microorganisms is an alternative to physical and chemical methods, and it can be easier, simpler, and without dangerous chemicals, economical, safe, clean, controlled size, and to be produced environmentally friendly in high yield (78, 97–99).

Vijayakumar et al. (33) presented the perspective of the effects of zinc nano oxide on some vital parameters of broiler chickens and declared that the mentioned source has a very good potential as an alternative to other zinc sources. Nano-ZnO can increase the performance and health of broiler chickens. However, more studies are needed to maximize nutrient digestibility, meat production, and bird health. Detailed mechanism interpretation is needed for practical recommendations and definition of the ideal level in the diet the applications of broiler chickens are still an urgent need for future research (33).

To provide a comprehensive overview of the currently available evidence, the findings of experimental studies evaluating the effects of nano zinc supplementation on productive performance, blood biochemical indices, antioxidant and immune responses, intestinal health, and tissue characteristics are summarized in Tables 2–5.

**Table 2:** Research on the effects of nano zinc consumption on the performance of broiler chickens.

Study Reference	Chicken strain	Age	Period time	Dose	Additive zinc	Challenge	Feed intake	Weight gain	FCR	Summary
Hussan et al. (120)	Commercial broiler	1–42 days	42 days	2.5, 5, 10 and 20 ppm Nano-ZnO + 40 ppm ZnO	Nano ZnO	None	↑	↑	↓	The dose of 2.5 ppm produced the best growth performance and FCR.
Ahmadi et al. (103)	Ross 308	Starter (1–21 days)	21 days	30, 60, 90 and 120 mg/kg	Nano ZnO	None	↓ (at 60 and 90 mg/kg)	↑	↓ (at 60 mg/kg)	Growth performance improved in the range of 30–90 mg/kg; the best FCR was observed at 60 mg/kg.
Zhao et al. (46)	Ross 308	1–42 days	42 days	20, 60 and 100 mg/kg	Nano ZnO	None	↔	↑ (20 and 60 mg/kg)	↓ (20 and 60 mg/kg)	20 mg/kg was identified as the optimal dose; 100 mg/kg reduced growth after day 28.
Chand et al. (7)	Hubbard (male)	7, 14, 21 and 35	35 days	50 ppm organic zinc + grape seed powder	Zinc-glycine (organic zinc, not nano zinc)	*Eimeria tenella* challenge	↑ (relative to positive control)	↑	↓	Improved performance under coccidiosis challenge; this study is not related to nano zinc.
Fathi et al. (47)	Ross 308	1–42 days	-	10, 20 and 40 mg/kg	Nano ZnO	None	↑	↔	↓	The use of 20 mg/kg of nano zinc oxide in the diet of broiler chickens was able to improve growth performance.
Dosoky et al. (100)	Cobb	1–35 days	35 days	5, 10, 20, 40, 60 and 80 ppm	Nano ZnO	Heat stress	↔	↔	↓	No significant reproductive effect was observed; immune and tissue responses were dose-dependent, with higher doses producing toxic effects.
Hatab et al. (98)	Cobb 500	3–35 days	5 weeks	40 and 60 mg/kg	Nano ZnO Bio (fungal)	None	↑	↑	↓	40 mg/kg produced the best production response and was introduced as the recommended dose.
Alian et al. (118)	Ross 308	1–35 days	35 days	40 mg Zn/kg	Nano ZnO	None	↓	↑	↓	Nano zinc produced the best growth performance compared to inorganic and organic sources of zinc.
Mohammed et al. (115)	Ross 308	1–42 days	42 days	6, 8, 10 and 12 mg/L drinking water Nano ZnO	Nano ZnO	None	↓	↑	↓	Addition of nano zinc to drinking water improved intestinal growth and morphology.
Abdel-Wareth et al. (109)	Ross 308	1–42 days	42 days	20, 40 and 60 mg/kg	Nano ZnO	Heat stress	↔	↑	↓	All levels of nano zinc improved growth performance and FCR under hot weather conditions.

FCR, Feed Conversion Ratio.

**Table 3:** Research on the effects of nano zinc consumption on the carcass traits of broiler chickens.

Reference	Dose	Carcass performance (% carcass)	Organ weight	Abdominal fat	Growth performance	Biological significance
Ahmadi et al. (103)	30, 60, 90 and 120 mg/kg	Not reported	Not reported	Not reported	↑ Body weight gain (at all doses) ↓ Feed intake (at doses 60 and 90) ↓ Feed conversion ratio (at dose 60)	Biologically meaningful improvement in growth performance at doses of 30 to 90 mg.
Mohammadi et al. (116)	80 (various types of zinc)	↓ in the nano-sulfate and control groups	Not reported	↑ In the Nano-Sulfate group	↓ Weight gain and feed intake in the nano-sulfate group	Negative effect of nano-sulfate on performance and increase in abdominal fat is biologically meaningful.
Abdel-Wareth et al. (109)	20, 40 and 60 mg/kg	↑ Increase in carcass percentage (linear effect)	Improved liver and kidney function (increased enzymes)	↓ Decrease in abdominal fat percentage (linear effect)	↑ Weight gain and decreased feed conversion ratio (linear effect)	Improved performance, increased carcass weight and reduced abdominal fat at all doses were biologically meaningful.

**Table 4:** Research on the effects of nano zinc consumption on some blood serum parameters of broiler chickens.

Reference	Dose	ALT	AST	Cr	UA	TP	TC	AOs	Inflammatory cytokines	Serum zinc concentration
Mohammed et al. (115)	6, 8, 10 and 12 mg/L (water)	↓	↓	-	-	No effect	-	Positive effect on antioxidants	-	-
Mahmood et al. (104)	40 mg/kg	↓	↓	-	-	↑	-	↑ GSH ↓ MDA	-	-
Dosoky et al. (100)	5, 10, 20, 40, 60 and 80 ppm	↓	↓	No effect	No effect	No effect	No effect	Dose-dependent changes	↑ Expression of inflammatory cytokines (at high doses)	-
Hatab et al. (98)	40 and 60 mg/kg	↑	↑	-	-	-	↓	-	-	↑ Zinc accumulation in serum, liver and muscle
Ibrahim et al. (117)	5, 10, 20, 40, 60 and 80 ppm	No effect	No effect	-	-	-	↓	Dose-dependent changes	↑ Expression of inflammatory cytokines (at high doses)	-
Fathi et al. (47)	10, 20 and 40 mg/kg	No effect	No effect	No effect	No effect	No effect	-	At 20 mg dose: ↑ T TAC, ↑ SOD, ↑ GPx, ↓ MDA	-	-

ALT, Alanine Aminotransferase; AST, Aspartate Aminotransferase; Cr, Creatinine; UA, Uric Acid; TP, Total Protein; TC, Total Cholesterol; AOs, Antioxidants; GSH, Reduced Glutathione; MDA, Malondialdehyde; TAC, Total Antioxidant Capacity; SOD, Superoxide Dismutase; GPx, Glutathione Peroxidase.

**Table 5:** Summary of the effect of different levels of nano zinc oxide consumption on carcass parameters, muscle tissue, and mineral deposition in poultry.

Reference	Nano zinc oxide consumption level	Approximate meat composition	Carcass traits	Muscle tissue characteristics	Deposition of minerals in tissue	Overall results
Dosoky et al. (2022) (100)	10, 20, 40, 60 and 80 ppm	No significant change	Not studied	Dose-dependent changes in pectoral muscle structure; muscle fiber destruction, mitochondrial damage and nanoparticle accumulation at high doses	Not studied	Consumption above 10 ppm caused structural changes in muscle, while lower doses were safer.
Abdel-Wareth et al. (2022) (109)	20, 40 and 60 mg/kg	Not studied	Increased carcass percentage and reduced abdominal fat	Not studied	Not studied	Zinc oxide nanoparticle improved carcass traits under heat stress conditions.
Ibrahim et al. (2017) (117)	50 mg/kg	No significant change	Not studied	Not studied	Increased zinc deposition in tibia and increased iron and copper in liver; no significant change in muscle minerals	ZnO nanoparticle increased zinc bioavailability, but did not significantly change muscle mineral composition.

Overall, the available evidence indicates that the biological effects of nano zinc are multifactorial and may depend on the zinc formulation, particle characteristics, supplementation level, diet composition, and experimental conditions. Selected studies investigating additional physiological responses are discussed below.

In addition to the reports related to the effects of nano zinc consumption, we can refer to the report of Dosoky et al. (100), who investigated the relative weight of the gizzard and intestine of broiler chickens in response to the consumption of Nano-ZnO supplements at doses of 5, 10, 20, 40, 60 and 80 mg/kg of feed. They found that they had decreased relative weights at feed concentrations of 40 and 60 mg/kg, with relative weights of kidney, calculus, and intestine decreased, and liver weight increased (98). Reda et al. (101) showed that with a decrease in the level of Nano-ZnO in the diets, a decrease in the relative weight of the intestine was observed, and following this event, the possibility of increasing the excretion and the activity of the endogenous enzyme in the intestine was enhanced due to the support of cofactors for several related metalloenzymes with better production of digestive enzymes (102). In addition, zinc can have a positive role on the intestinal health of broiler chickens by improving intestinal integrity, crypt cell function, barrier structures, and protein synthesis (98). In the reports, the increase in liver weight is also attributed to the increase in the bioavailability of nanoparticles, which is due to the retention of zinc in the liver after absorption (103). Mahmood et al. (104) also found that the relative weight of the bursa and spleen in chickens that consumed diets containing 10, 20, 30, or 40 mg/kg of zinc oxide had a significant increase compared to the control group. This was thought to indicate an increase in thymulin activity and the maturation of T lymphocytes, as well as the activation of B lymphocytes by T-helper cells, and finally the improvement of the immune responses of chickens (105). El-Maddawy et al. (106) reported that supplementation of broiler chickens’ diets with Nano-ZnO at the level of 5 to 80 mg/kg was associated with an increase in hematocrit, hemoglobin, and white blood cell percentage. It is hypothesized that the role of zinc in erythropoiesis is important for the production of red blood cells and hemoglobin (107). In addition, the role of zinc in the catalysis and activation of alpha-aminolaevulinic acid dehydrogenase is very important for the synthesis of heme (108). Abdel-Wareth et al. (109). By feeding 20, 40, and 60 mg/kg of Nano-ZnO in birds, they were able to show a decrease in the serum levels of uric acid and creatinine, and their reduction indicates the health of this body organ. Studies have confirmed the role of zinc in oxidative defense (97, 110). The mechanism includes increasing SOD, glutathione peroxidase (GPx), and total antioxidant capacity (TAC), and reducing the concentration of malondialdehyde (MDA), improving the immune response, and maintaining the redox balance of the bird’s immune system by removing reactive oxygen species (ROS).

In the report of Reda et al. (101), the decrease in the number of *Escherichia coli*, *Salmonella*, and *Enterococcus* bacteria and the increase of beneficial intestinal *Lactobacillus* bacteria with Nano-ZnO supplementation at levels of 0.1 and 0.3 g/kg were significant (74). However, the antibacterial property of nanoparticles is mainly inversely correlated with the particle size, and the smaller the particle, the higher the ratio of specific surface area to volume, and subsequently, the availability of the material will be better (23).

Antimicrobial mechanisms of Nano-ZnOs are through the electrostatic connection of nanoparticles with the bacterial cell membrane. In fact, negatively charged microorganisms and positively charged metal oxides are connected to each other, and this interaction has caused disruption and deformation of the integrity of the cell membrane in a number of bacteria, which can lead to the loss of membrane permeability and transport. This means the nanoparticle can be transported more easily throughout the cell (97). When Nano-ZnO penetrates bacteria, compounds containing phosphorus and sulfur, such as deoxyribonucleic acid (DNA) of bacteria, react and bactericidal activity occurs (111, 112). In addition, by preventing the growth of a wide range of pathogens and strengthening the non-specific function against bacteria, Nano-ZnOs play an indirect role similar to antibiotics and will be excellent candidates without the risk of bacterial resistance (97, 113, 114). To facilitate comparison across studies, the available evidence regarding the effects of nano zinc supplementation on intestinal health, antimicrobial activity, and related physiological responses in broiler chickens is summarized in Table 6.

**Table 6:** Researches on the effects of nano zinc consumption on nutrient digestibility.

Reference	Dose (mg/kg diet)	Effect on crude protein digestibility	Effect on crude fat digestibility	Effect on crude fiber digestibility	Key point
Dosoky et al. (100)	10, 20, 40, 60 and 80 ppm	No significant effect	No significant effect	No significant effect	In summer (heat stress); high doses (≥40) cause tissue damage.
Abdel-Wareth et al. (109)	40 and 60 mg/kg	Significant increase (linear)	Significant increase (linear)	Significant increase (linear)	In warm weather conditions; performance and digestibility improvements were observed together.
Ibrahim et al. (117)	50 mg/kg	No significant effect	Significant increase (86.7%)	No significant effect	Comparing different zinc sources; nanoparticles and nano+organic mixture had the best results for fat.

In most of the studies mentioned, the improvement in growth performance has been attributed to increased zinc bioavailability. Reducing the particle size increases the specific surface area and greater contact with the intestinal mucosa, facilitating more effective dissolution of nanoparticles, gradual release of Zn^2+^ ions, and increased absorption through specific zinc transporters. Zinc transporters are a set of membrane proteins that regulate the absorption, transport, distribution, and maintenance of homeostasis of zinc ions (Zn^2+^) in cells. Since zinc ions cannot freely cross the cell membrane, their entry and exit are completely dependent on these specific transporters. In broiler chickens, two main families of zinc transporters are known, namely ZIP (Zrt-/Irt-like Proteins; SLC39) and ZnT (Zinc Transporters; SLC30), which can increase intracellular zinc concentration. The functions of ZIP include the transport of Zn^2+^ from the intestinal lumen into the intestinal epithelial cells and the transport of zinc from the extracellular space or intracellular organelles to the cytoplasm. In other words, ZIPs are responsible for the entry of zinc into the cell. In the intestine, the most important member of this family is ZIP4, which plays a major role in the absorption of dietary zinc. When zinc stores in the body decrease, the expression of the ZIP4 gene increases to increase zinc absorption. Also, the ZnT transporter reduces the concentration of zinc inside the cell. Its function is to: transport zinc from the cytoplasm to the outside of the cell and transport zinc into organelles such as the Golgi apparatus, endoplasmic reticulum, and vesicles for storage or metabolic use. As a result, ZnTs are responsible for the exit or storage of zinc. In intestinal cells, ZnT1 is the most important transporter for the exit of zinc from the enterocyte to the bloodstream. It should be noted that metallothionein, along with ZIP and ZnT, plays a role in regulating zinc homeostasis by temporarily binding excess zinc, preventing toxicity from excessive Zn^2+^, and releasing zinc ions again when needed. Therefore, metallothionein acts as a regulatory zinc reservoir within the cell. It is hypothesized that after poultry consume Nano-ZnO, some of the nanoparticles dissolve in the acidic environment of the digestive tract and release Zn^2+^ ions. These ions enter the intestinal epithelial cells via ZIP transporters. Then, some of the zinc is stored by metallothionein, and the other part is transported to the bloodstream or other organs via ZnT transporters. Increasing the bioavailability of Nano-ZnO improves the regulation of this zinc transport system, and as a result, supports and enhances the activity of zinc-dependent enzymes, antioxidant responses, immune function, and intestinal health. Indeed, the coordinated activity of these transporters, together with metallothionein, regulates intracellular zinc concentration and influences bioavailability, antioxidant responses, immune function, and intestinal health (59, 74, 81, 98, 115, 116).

In addition, a review of the aforementioned studies showed that the activity of many metalloenzymes, protein synthesis, cell proliferation, and secretion of growth-related hormones, including Insulin-like Growth Factor 1 (IGF-1) and growth hormone, were improved, and these processes ultimately led to increased body weight, improved feed conversion ratio (FCR), and increased carcass yield (50, 57, 69, 79, 117, 118). On the other hand, improved growth performance is not only a consequence of increased zinc absorption, but also the health of the digestive tract plays a key role in this process. Studies show that Nano-ZnO increases villus height, reduces crypt depth, increases the villus height to crypt depth ratio, and enhances the integrity of epithelial tight junctions. These changes increase the level of nutrient absorption, reduce intestinal permeability, and prevent the transmission of endotoxins and pathogenic bacteria. In addition, zinc, as a cofactor for many digestive enzymes, also enhances crypt cell activity and the synthesis of epithelial structural proteins, which together improve digestibility and nutrient utilization (59, 72, 74, 81, 98, 105, 115). At the same time, Nano-ZnO also plays a role in improving performance by modulating the composition of the intestinal microbiota. Most studies have reported a decrease in the population of pathogenic bacteria such as *Escherichia coli and Salmonella* and a relative increase in beneficial bacteria such as *Lactobacillus* and butyrate producers from the *Lachnospiraceae* family. Increased production of SCFA, especially butyrate, provides the energy needed by intestinal epithelial cells, inhibits inflammation, and increases resistance to colonization by pathogens. In addition to the direct effects of zinc ions on bacterial membranes, ROS production, Zn^2+^ ion release, and disruption of bacterial DNA and protein function also contribute to the antibacterial properties of nanoparticles (73, 74, 81, 90, 93–96, 111, 112).

Enhancement of the immune response was also another major mechanism of action of Nano-ZnO in research. Increased thymulin activity, T lymphocyte development, B lymphocyte stimulation, increased macrophage and heterophil activity, and increased immunoglobulin production are among the most important changes reported. In addition, molecular studies have shown that Nano-ZnO reduces the severity of systemic inflammation by inhibiting the activation of the Nuclear Factor Kappa B (NF-κB) pathway and reducing the production of proinflammatory cytokines, while simultaneously increasing the antioxidant defense capacity. Increased SOD, GPx, and TAC activity, along with decreased MDA, indicate maintenance of redox balance and reduction of damage caused by reactive oxygen species (47, 79, 82, 85, 97, 106, 110, 118).

Despite this promising evidence, not all studies have reported the same responses. Some trials have shown only limited improvements in growth performance or safety indices, and in some cases no significant difference was observed compared to conventional zinc sources. This heterogeneity is likely due to differences in the characteristics of the nanoparticles, including particle size (e.g., 21 vs. 82 nm), synthesis method (chemical, physical or biological), surface coating, release rate of Zn^2+^ ions, dose, initial dietary zinc status, age and physiological status of the birds, stress or disease challenge conditions, and duration of administration. In many studies, smaller nanoparticles have been shown to have higher biological activity due to their larger surface area, but this same property can increase the likelihood of tissue accumulation and toxicity at high doses (Table 7) (80, 81, 97, 98, 100, 109, 113, 119).

**Table 7:** Comparison of different sources of zinc supplementation used in broiler feeding in terms of dosage, bioavailability, tissue retention, toxicity and economic considerations.

Zinc source	Dose (mg/kg)	Bioavailability	Appearance of zinc digestibility	Zinc retention in the body	Fecal zinc excretion	Safety and potential for tissue accumulation	Zinc excretion rate	Environmental impact	Regulatory status	Economic considerations	Most important feature	Reference
Zinc sulfate (ZnSO₄)	40–100	Moderate (16, 50, 58, 80)	Moderate (16, 50, 58, 80)	Moderate (16, 50, 58)	High (16, 50, 58, 80)	Increased excretion and environmental contamination at high doses	Low	Relatively high	Permitted	Cheap and available	Common mineral source	(16, 50, 58, 80)
Zinc Oxide (ZnO)	40–100	Low to moderate (16, 58, 80)	Low to moderate (16, 58, 80)	Low to moderate (16, 58, 80)	High (16, 58, 80)	Limited bioavailability; more required to meet needs	Low	High	Limited in some countries	Cheap	Less effective than other sources	(16, 58, 80, 125)
Zinc Hydroxychloride	30–80	High (81, 97, 98, 100, 120)	Moderate to high (81, 97, 98, 100, 120)	Moderate (81, 97, 98, 100, 120)	Moderate (81, 97, 98, 100, 120)	High safety, moderate accumulation	Moderate to high	Moderate permissible	مجاز	Economically justified	Good adsorption capacity More stable, less reactivity	(80, 81, 87, 97, 98, 100, 109, 113, 120)
Coated ZnO	20–60	High (80, 87, 124)	High (80, 87, 124)	Medium (80, 87, 124)	Medium (80, 87, 124)	High safety and moderate accumulation	High	Low	Under development	Available and economically justified	Controlled release in the intestine	(80, 87, 124)
Potentiated ZnO	20–60	High (80, 87, 124)	High (80, 87, 124)	Medium (80, 87, 124)	High	High safety and moderate aggregation	High	Low	Under development	More available and cost	Increase efficiency by structural modification or additives	(80, 87, 124)
Organic sources of zinc (Zn-methionine, Zn-glycine, Zn-proteinate, and others)	30–80	High (50, 123, 126)	High (50, 123, 126)	High (50, 123, 126)	Lower than mineral sources (50, 123, 126)	Good safety; low toxicity	High	Low	Permissible	Higher cost	Higher and better absorption and reduced excretion of zinc	(50, 123, 125, 126)
Zinc oxide nanoparticles (Nano-ZnO)	20–60	Very high (81, 97, 98, 109, 113, 120)	High (81, 97, 98, 109, 113, 120)	High (81, 97, 98, 109, 113, 120)	Low (81, 97, 98, 109, 113, 120)	Safe at recommended doses; at high doses, accumulation in liver, kidney, and bone is possible	Very high	Very low	Needs further investigation	Higher production cost but lower consumption	Increased bioavailability, reduced need for supplementation, highest absorption	(81, 97, 98, 109, 113, 120, 125)
Reinforced Porous Zinc Oxide (PP-ZnO)	30–60	High (124)	High (124)	High (124)	Low (124)	Good safety	High	Low	Acceptable	Moderate cost	Maintains intestinal health and reduces zinc excretion without nanotechnology	(124, 125)
Encapsulated formulations or hybrid zinc delivery systems	Under investigation	Probably high (13, 123–127)	Limited data (13, 123–127)	Limited data (13, 123–127)	Probably low (13, 123–127)	Needs further evaluation	Probably high	Probably low	Not yet commercialized	Not yet commercialized	Next generation zinc supplements	(13, 123–128)

The qualitative descriptors (high, medium, and low) presented in table represent relative rankings derived from published comparative studies on bioavailability, digestibility, tissue retention, zinc excretion, and production performance. These descriptors do not represent absolute quantitative threshold.

In this regard, toxicity studies have shown that the response to Nano-ZnO is dose-dependent and that excessive increases in supplementation levels are not necessarily associated with greater improvement. Moderate doses (around 20–40 mg/kg in most studies) have generally provided the best balance between increased bioavailability and safety, whereas higher doses may be associated with zinc accumulation in the liver and kidneys, changes in the relative weight of some organs, increased oxidative stress, and disruption of trace element homeostasis. Therefore, the main advantage of nanotechnology is not in increasing the dose, but in increasing bioavailability and reducing the amount of supplementation required (97, 98, 100, 109, 113, 120).

## Comparison of chemically and biologically synthesized nano zinc

ZnO nanoparticles (ZnO-NPs) used in poultry feed are mainly produced through two approaches: chemical synthesis and biosynthesis (green). In the chemical synthesis method, inorganic precursors, reducing agents, and chemical stabilizers are used to produce nanoparticles with uniform size, high purity, and industrial scale production capability. This method allows for precise control of the size, morphology, and physicochemical properties of the nanoparticles, but is usually associated with higher energy consumption, the use of potentially toxic chemicals, and the generation of hazardous wastes, which can raise concerns in terms of biosafety and environmental consequences (119, 121).

In contrast, the biosynthesis or green synthesis of zinc oxide nanoparticles is carried out using extracts of plants, bacteria, fungi, or other biological agents. In this method, bioactive compounds present in biological resources, in addition to playing the role of reducing and stabilizing agents, lead to the formation of nanoparticles with greater biocompatibility and reducing the need for hazardous chemicals. This technology is known as one of the most important approaches of green nanotechnology due to its lower energy consumption, reduced production of chemical pollutants and greater compatibility with the environment (119, 122).

In addition to environmental benefits, studies have shown that nanoparticles synthesized by biological methods have greater colloidal stability, higher biocompatibility and lower toxicity than nanoparticles synthesized by chemical methods due to the presence of a layer of biological compounds on the surface of the particles. These features can increase the bioavailability, cellular uptake and bioefficiency of nanoparticles and reduce the risk of oxidative stress caused by nanoparticle aggregation (121, 122).

In the field of poultry nutrition, both types of zinc oxide nanoparticles have been able to improve growth performance, antioxidant status, immune response, gut health, and nutrient efficiency. However, biosynthesized nanoparticles have been proposed as a promising option for the development of sustainable and environmentally friendly feed additives due to their lower toxicity, greater biocompatibility, and better stability. However, standardization of synthesis processes, control of particle size and morphology uniformity, reproducibility of production on an industrial scale, and comprehensive long-term safety assessment remain the most important challenges for the widespread application of biosynthesized zinc oxide nanoparticles in the poultry industry (119, 121, 122).

## New zinc supplement technologies; beyond zinc oxide nanoparticles

In recent years, the development of new trace element supplementation technologies has shown that zinc oxide nanoparticles are not the only solution available to increase zinc bioavailability. Advances in materials engineering and mineral element delivery technologies have enabled the design of a new generation of zinc supplements that, by modifying physicochemical properties, increasing specific surface area, improving solubility, and controlling the release of zinc ions, increase the absorption of this element while reducing zinc excretion into the environment (123, 124). One of the most recently introduced technologies is potentiated porous zinc oxide, which increases the bioavailability of zinc ions compared to conventional zinc oxide by increasing the porosity and contact surface of the particles. In a study by Marchetti et al. (124), the use of this innovative zinc source in the diet of newly weaned piglets resulted in the maintenance of growth performance, improvement of systemic and mucosal immune responses, reduction of intestinal permeability markers and favorable modulation of fecal microbiota. Although this study was conducted in the pig species, the reported mechanisms, including enhancement of intestinal barrier function, regulation of immune responses and reduction of inflammation, are consistent with the known mechanisms of zinc oxide nanoparticles in poultry and can be a good basis for the development of future research in broilers (124). In addition to particle-based technologies, organic zinc chelates are also considered one of the most successful strategies to increase zinc bioavailability. In these compounds, the binding of zinc ions to organic ligands such as amino acids or proteinates increases the stability of zinc in the digestive tract, reduces its competition with other mineral elements, and thus provides more effective absorption. Several studies have shown that the use of organic sources of zinc can improve growth performance, immune response, and zinc retention, while simultaneously reducing zinc excretion through feces (123). In recent years, other technologies such as microencapsulation, controlled-release zinc delivery systems, and other engineered mineral delivery systems have also received attention. The main goal of these technologies is to protect zinc compounds during passage through the digestive tract, gradually release zinc ions at the appropriate site of absorption, reduce interactions with other dietary components, and ultimately increase the bioavailability of the supplement. Although most of these technologies are still in the research and development stages and evidence from poultry studies is limited, preliminary results suggest that these systems could be used as alternatives or complements to nanotechnology in the future (124). Overall, recent research trends suggest that the future of poultry mineral nutrition is likely to be based on the use of a new generation of zinc supplements with high bioavailability, targeted release, reduced supplement consumption, and minimal environmental disposal. Among them, zinc oxide nanoparticles remain one of the most important technologies under study, but emerging technologies such as reinforced porous zinc oxide, organic zinc complexes, and advanced zinc delivery systems also offer promising prospects for improving intestinal health, increasing absorption efficiency, and promoting environmental sustainability in the poultry industry (123–125).

## Molecular mechanisms of the effect of nano-zinc in broiler chickens

### Cellular uptake of nano-zinc, zinc homeostasis, and the role of ZIP/ZnT transporters

Although increased zinc bioavailability is considered one of the most important advantages of using zinc oxide nanoparticles (Nano-ZnO), the molecular mechanisms responsible for this increased bioavailability have received little attention until recent years. Unlike conventional mineral sources, zinc oxide nanoparticles establish more effective contact with the intestinal mucosa after entering the gastrointestinal tract due to their small size, large specific surface area and higher reactivity. Some of these particles gradually dissolve in the acidic environment of the gizzard and the initial part of the small intestine and release Zn^2+^ ions, while another part can directly enter the intestinal epithelial cells through endocytosis or vesicle-dependent pathways. The presence of these two uptake routes increases the bioavailability of nano-zinc compared to conventional sources and allows for more effective use of smaller amounts of supplementation (50, 57, 79, 98, 117). Once zinc enters the cell, its cytosolic concentration must be carefully regulated, as both a deficiency and an excess of Zn^2+^ ions can disrupt normal cell function. This homeostasis is mainly controlled by two families of zinc transporter proteins, namely ZIP and ZnT. As mentioned, ZIP transporters facilitate the transport of zinc from the extracellular space or intracellular organelles to the cytoplasm, while ZnT transporters are responsible for the exit of zinc from the cytoplasm or its transport to organelles such as the endoplasmic reticulum and Golgi apparatus. This two-way regulation maintains optimal zinc concentrations for the activity of enzymes, regulatory proteins, and cellular signaling pathways (50, 78, 79, 97, 114, 119, 121–125). Evidence suggests that increased zinc bioavailability after Nano-ZnO administration can modulate the expression of some intestinal transporters, particularly ZIP4 and ZnT1, in accordance with the nutritional status of the bird. Under conditions of zinc deficiency, the expression of ZIP transporters is increased and zinc absorption is enhanced, while under conditions of increased zinc intake, the expression of ZnT transporters and other cellular excretory pathways is increased, preventing excessive zinc accumulation. This adaptive response is considered to be an important factor in reducing the risk of toxicity at appropriate doses of nano-zinc, although at very high levels the capacity of these regulatory systems may be saturated and cellular homeostasis may be disrupted (79, 97, 98, 100). Fine-tuning of zinc transport is not only essential for maintaining the balance of this element, but also initiates many subsequent biological responses. Increased intracellular Zn^2+^ concentrations affect the activity of hundreds of metalloenzymes, transcription factors, and signaling proteins, and provide the basis for the activation of antioxidant pathways, immune regulation, cell proliferation, and repair of the intestinal epithelium. Therefore, ZIP and ZnT transporters can be considered as the starting point of a complex network of molecular responses that ultimately lead to improved growth performance and health of broilers (50, 79, 98, 117, 119, 121–127).

### Regulation of metallothionein expression and intracellular zinc homeostasis

After zinc ions enter the cell, a significant portion of them are bound by metallothioneins. As mentioned, in addition to storing and transporting zinc, this group of compounds plays an important role in regulating metal homeostasis, detoxifying heavy metals, and protecting cells against oxidative stress. Due to the large number of sulfhydryl (-SH) groups, these proteins have a high ability to bind Zn^2+^ ions and also neutralize reactive oxygen species, and for this reason they are considered one of the first lines of defense of the cell against oxidative damage (78, 79, 85, 97). The expression of metallothionein genes is mainly under the control of the transcription factor Metal-responsive Transcription Factor-1 (MTF-1). The increase in intracellular Zn^2+^ concentration after Nano-ZnO administration activates MTF-1 and its binding to metal response elements (MREs) in the promoter region of MT genes. As a result, metallothionein synthesis is increased and the cell’s capacity to store and regulate zinc is enhanced. This response not only prevents excessive free zinc, but also provides an accessible zinc pool for enzymatic reactions and metabolic processes (79, 85, 97). In addition to their regulatory role, metallothioneins are also directly involved in reducing oxidative stress. The thiol groups present in the structure of these proteins are able to neutralize reactive oxygen and nitrogen species and prevent the oxidation of proteins, lipids, and DNA. This function, along with the activation of the nuclear factor erythroid 2-related factor 2 (Nrf2) pathway, enhances the antioxidant capacity of the cell and reduces damage caused by environmental stresses such as heat stress, infectious challenges and high density (79, 82, 85, 106, 125). On the other hand, overexpression of metallothionein at high doses of nano zinc can act as a negative feedback mechanism. In this situation, the binding of a large part of the zinc ions to metallothionein reduces free cytosolic zinc and limits its availability for some metabolic processes. This is probably one of the reasons for the observation of dose-dependent responses in some studies, in which further increases in nano zinc levels were not associated with further improvements in growth performance (13, 97, 98, 100, 109, 123–127).

### Activation of the Nrf2 antioxidant pathway and regulation of oxidative stress

One of the most important molecular mechanisms that can explain a significant part of the beneficial effects of Nano-ZnO is the activation of the Nrf2 signaling pathway. This pathway is known as a master regulator of cellular antioxidant responses and plays a pivotal role in maintaining the redox balance, reducing oxidative damage, and increasing cellular resistance to stressors. Under normal conditions, the Nrf2 protein is bound in the cytoplasm by the Keap1 protein and is continuously targeted for proteasomal degradation. However, a mild increase in reactive oxygen species or changes in intracellular Zn^2+^ concentration can disrupt this binding and cause Nrf2 release (79, 82, 85, 106, 110). A review of the scientific literature has shown that, upon release, Nrf2 translocates to the cell nucleus and binds to antioxidant response elements (AREs), increasing the expression of a wide range of protective genes. These genes include enzymes such as SOD, GPx, CAT, co-oxygenase-1 (HO-1), and NAD(P)H quinone oxidoreductase-1 (NQO1), which play a key role in scavenging reactive oxygen species and maintaining cellular redox balance. Increased activity of these enzymes has been reported in many nutritional studies in association with decreased MDA concentration and increased TAC (47, 79, 82, 85, 106, 110, 118). In addition to reducing oxidative damage, activation of the Nrf2 pathway also supports normal mitochondrial function, intestinal epithelial cell proliferation, and damaged tissue regeneration. As a result, reduced oxidative stress maintains intestinal mucosal integrity, increases nutrient absorption, and reduces energy expenditure for inflammatory responses. These changes ultimately explain part of the improved growth performance, FCR, and resistance of birds to environmental stresses (79, 82, 98, 106). However, the response of the Nrf2 pathway is also highly dose-dependent. While low to moderate doses of Nano-ZnO typically activate this pathway and increase antioxidant capacity, excessive consumption of nano-zinc may suppress the cell’s defense capacity and even induce oxidative stress due to intracellular accumulation and increased ROS production. This phenomenon is probably one of the main reasons for the difference in results of studies on antioxidant indices and indicates that the effects of nano zinc do not follow a linear relationship, but rather depend on the balance between increasing bioavailability and maintaining cellular homeostasis (13, 97, 98, 100, 109, 113, 124–127).

### Inhibition of the NF-κB pathway and modulation of inflammatory cytokine signaling

Regulation of inflammatory responses is one of the most important mechanisms through which Nano-ZnO can improve intestinal health and growth performance of broiler chickens. Under normal conditions, exposure to pathogens, bacterial lipopolysaccharides (LPS), heat stress or increased ROS activates Pattern Recognition Receptors, particularly Toll-like receptor 4 (TLR4). This process is accompanied by activation of the IκB kinase (IKK) complex, phosphorylation of the inhibitory protein or inhibitor of nuclear factor kappa B (IκB), and release of the transcription factor nuclear factor-kappa B (NF-κB). After NF-κB translocation to the nucleus, the expression of a wide range of proinflammatory genes, including Tumor Necrosis Factor-Alpha (TNF-*α*), Interleukin-1 beta (IL-1β), Interleukin-6 (IL-6), Interleukin-8 (IL-8), Cyclooxygenase-2 (COX-2), and Inducible Nitric Oxide Synthas (iNOS), is increased, and the inflammatory response is initiated (47, 79, 82, 85, 97, 106, 110). Studies show that Nano-ZnO can inhibit this pathway from several different points. First, increasing the bioavailability of zinc reduces the production of ROS in the cell and prevents the initial activation of the IKK complex. Second, zinc is able to maintain the stability of the IκB protein and prevent the translocation of NF-κB to the nucleus. Third, reducing oxidative stress and improving mitochondrial function reduces the production of secondary inflammatory messengers, and as a result, the expression of proinflammatory cytokines is reduced. These changes are associated with reduced inflammation of the intestinal mucosa, reduced tissue damage, and maintenance of normal epithelial function (79, 82, 85, 97). In contrast, Nano-ZnO, in addition to inhibiting proinflammatory cytokines, can also increase the expression of anti-inflammatory cytokines such as Interleukin-10 (IL-10) and Transforming Growth Factor-*β* (TGF-β). This change in cytokine balance limits the inflammatory response, accelerates tissue repair, and prevents chronic inflammation. Such a change is of particular importance in conditions of heat stress, microbial challenges, and coccidiosis, as uncontrolled inflammation is one of the main factors that reduce growth performance and increase feed conversion (79, 85, 106, 118). One of the most important aspects of Nano-ZnO function is the crosstalk between the Nrf2 and NF-κB pathways. These two regulatory pathways reciprocally affect each other’s activity. Activation of Nrf2 eliminates one of the most important triggers for NF-κB activation by increasing the expression of antioxidant enzymes and reducing ROS production. On the other hand, inhibition of NF-κB reduces the production of pro-inflammatory cytokines and reduces ROS production by immune cells, which helps maintain Nrf2 activity. Therefore, Nano-ZnO shifts the balance between oxidative and inflammatory responses toward cellular homeostasis by creating a positive cycle between Nrf2 activation and NF-κB inhibition. This interaction is likely one of the most important reasons for the simultaneous improvement of antioxidant capacity, intestinal health, and growth performance in many studies (79, 82, 85, 97, 106, 110, 125). However, the intensity of NF-κB inhibition has not been consistent across studies. Differences in nanoparticle size, surface properties, synthesis method, dosage, initial dietary zinc status, and severity of inflammatory challenge can influence the magnitude of the response of this pathway. In conditions where baseline inflammation is mild, the reduction in cytokine expression may be limited and not statistically significant, whereas in birds under stress or infection, the anti-inflammatory effects of Nano-ZnO have been reported to be more pronounced (13, 79, 97, 100, 109, 113, 125–127).

### Interaction of Nano-Zn with mucosal immune cells, Iga production, and mucosal immunity

The gastrointestinal tract is not only the primary site of nutrient absorption, but also the largest immune organ in the body. More than 70% of the body’s immune cells reside in the gut-associated lymphoid tissue (GALT), and maintaining a balance between tolerance to dietary antigens and response to pathogens is essential for normal gut health and function. Zinc is a key element in the development, differentiation, and function of these immune cells, and increasing its bioavailability after Nano-ZnO administration can affect many mucosal immune processes (47, 79, 85, 97, 106, 125–127). Studies have shown that Nano-ZnO improves the function of antigen-presenting cells such as macrophages and dendritic cells. These cells initiate specific immune responses by increasing phagocytosis, antigen processing, and controlled cytokine secretion. In addition, zinc is essential for the maturation of T lymphocytes in the thymus and the maintenance of helper Cluster of Differentiation 4^+^ (CD4^+^) and cytotoxic Cluster of Differentiation 8^+^ (CD8^+^) lymphocyte activity. Increased activity of thymulin, a zinc-dependent hormone, facilitates the normal development and function of T lymphocytes and enhances the cellular immune response (47, 79, 82, 106, 118). Nano-ZnO can affect the function of B lymphocytes and the production of immunoglobulins. In many studies, an increase in the concentration of secretory IgA (sIgA) in the intestinal mucosa has been reported after the administration of Nano-ZnO. IgA is the most important mucosal immunoglobulin and, by binding to bacteria, viruses, and microbial toxins, prevents them from binding to epithelial cells. This process, known as immune exclusion, constitutes the first line of defense specific to the intestinal mucosa and controls the microbial load without causing severe inflammation (79, 85, 106, 110, 118, 125). Increased IgA production is not only a direct result of B-lymphocyte stimulation, but also involves improved epithelial cell function, reduced inflammation, and altered microbiota composition. Reduced mucosal inflammation preserves the structure of intestinal lymphoid follicles and improves communication between epithelial cells, dendritic cells, and lymphocytes. As a result, the mucosal immune response is directed toward a regulated and effective response, rather than an intense inflammatory response, that controls pathogens while causing minimal damage to intestinal tissue (79, 85, 97, 106). However, the immune responses induced by Nano-ZnO have not been consistent across studies. Differences in bird age, flock health status, presence or absence of microbial challenge, diet composition and supplement dosage are the most important factors causing these differences. Especially in healthy birds where the immune system is in a state of equilibrium, the increase in immune indices may be limited, while in conditions of heat stress or infection, the immune response to Nano-ZnO is usually more pronounced (79, 97, 100, 109, 125).

### Microbiota–host immunity interactions, distinguishing direct and indirect antibacterial effects and the role of SCFA

One of the most important issues raised in recent studies on Nano-ZnO is the distinction between direct antimicrobial effects and indirect effects resulting from improved host immunity and intestinal barrier function. In many reports, the reduction of pathogenic bacterial populations has been attributed solely to the antibacterial properties of nanoparticles, while existing evidence suggests that a significant part of these changes is the result of simultaneous improvement of epithelial function, modulation of the immune response, and creation of a more favorable environment for the growth of beneficial bacteria (73, 74, 81, 90, 93–96, 111, 112, 125, 126).

In terms of direct effects, Nano-ZnO can inhibit bacterial growth by several mechanisms. The release of Zn^2+^ ions disrupts the function of membrane proteins, increases membrane permeability, disrupts ionic balance, and inhibits vital enzymes. In addition, the production of reactive oxygen species in the vicinity of the nanoparticle surface increases oxidative damage to cell membranes, DNA, and bacterial proteins, ultimately inhibiting the growth or death of microbial cells. These effects have been reported to be particularly pronounced against Gram-negative bacteria such as *Escherichia coli* and Salmonella (73, 90, 93–96, 111, 125). In contrast, a significant part of the microbiota changes are likely due to indirect effects of Nano-ZnO. Improved epithelial barrier integrity, reduced inflammation, and increased IgA production provide a more stable environment for the growth of symbiotic bacteria. As a result, the population of beneficial bacteria such as *Lactobacillus*, *Bifidobacterium*, and some members of the *Lachnospiraceae* family increases and biological competition with pathogens is enhanced. This process, known as colonization resistance, is one of the most important mechanisms of natural control of pathogens in the intestine and prevents the establishment of pathogenic microorganisms without the need for direct antibacterial activity (74, 81, 93, 95, 111, 125).

Altering the composition of the microbiota also has important metabolic consequences. The increase in fiber-fermenting bacteria leads to increased production of SCFAs, including acetate, propionate, and especially butyrate. Butyrate is the main energy source for colonic epithelial cells and, in addition to providing energy, also promotes the expression of tight junction proteins, epithelial cell proliferation, mucosal repair, and differentiation of regulatory T lymphocytes (Treg). SCFAs also help maintain mucosal immune homeostasis and reduce chronic inflammation by inhibiting the NF-κB pathway and increasing IL-10 production. Therefore, part of the beneficial effects of Nano-ZnO on growth performance may be due to functional changes in the microbiota and its metabolites, rather than simply changes in the relative abundance of bacterial species (74, 81, 93–96, 111, 112, 125). Based on the available evidence, an integrated mechanistic model for the action of Nano-ZnO in the intestine can be proposed (Figure 3). In this model, Nano-ZnO improves zinc homeostasis after entering the gastrointestinal tract by controlled release of Zn^2+^ ions and increased uptake by ZIP transporters. Intracellular zinc enhances antioxidant capacity and immune balance by activating the Nrf2 pathway, increasing metallothionein expression, and inhibiting the NF-κB pathway. At the same time, the preservation of tight junction proteins, reduced intestinal permeability, and increased IgA production strengthen the mucosal barrier. These changes, together with the modulation of microbiota composition and increased production of SCFAs, create a stable environment for normal intestinal function, ultimately leading to improved digestibility, more efficient nutrient utilization, reduced energy expenditure for inflammatory responses, and improved growth performance in broiler chickens. This model suggests that the effects of Nano-ZnO result from the interaction of multiple molecular and physiological pathways and cannot be limited to antibacterial properties or increased zinc absorption alone (33, 50, 78, 79, 97, 106, 111, 113, 125).

**Figure 3:**
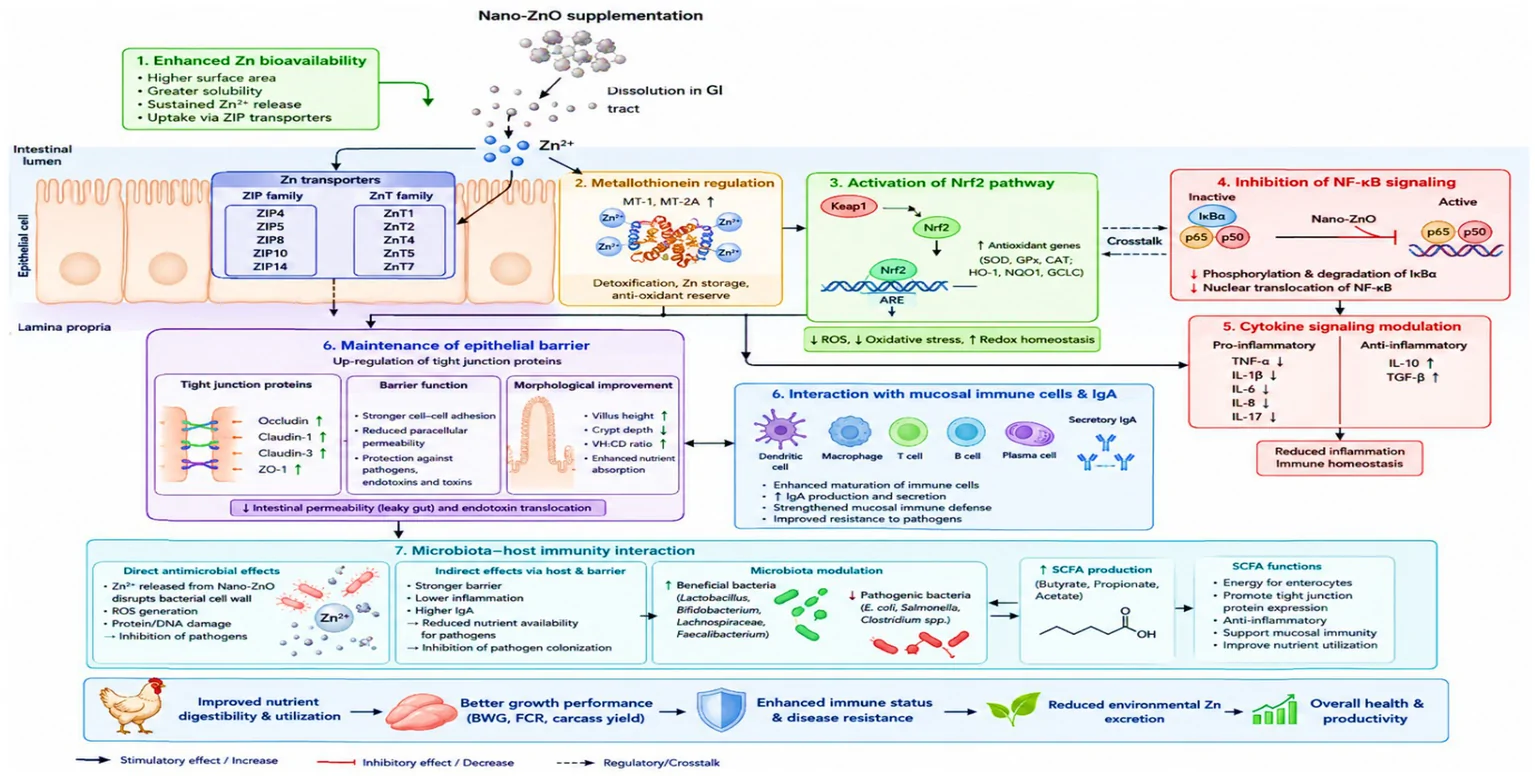
Schematic view of the mechanistic model of the effects of zinc oxide nanoparticles (Nano-ZnO) supplementation on zinc homeostasis, intestinal health, antioxidant responses, immune regulation, and production performance of broiler chickens. Zn, Zinc; ZIP, Zrt-, Irt-like Protein; ZnT, Zinc Transporter; MT, Metallothionein; Nrf2, Nuclear factor erythroid 2-related factor 2; NF-KB, Nuclear factor kappa B; ROS, Reactive oxygen species; IgA, Immunoglobulin A; SCFA, Short-chain fatty acids.

### Maintaining the integrity of the intestinal epithelial barrier and regulating tight junction proteins

In addition to its role in the digestion and absorption of nutrients, the digestive tract is the body’s first line of defense against pathogens, toxins, and antigens in the intestinal contents. The normal function of this barrier depends on the health of epithelial cells, the integrity of intercellular junctions, and the balance between immune responses and the intestinal microbiota. Any disruption in these structures can lead to increased mucosal permeability (leaky gut), the passage of LPS, endotoxins, and other microbial products into the bloodstream, and ultimately lead to systemic inflammation, reduced nutrient absorption, and impaired growth performance. In recent years, zinc oxide nanoparticles (Nano-ZnO) have been considered as one of the most effective mineral supplements for maintaining intestinal epithelial barrier function due to their higher bioavailability and ability to regulate multiple cellular pathways (13, 125–127). One of the most important protective mechanisms of Nano-ZnO is the regulation of the expression and stability of tight junction proteins, including Occludin, Claudin-1, Claudin-3, and Zonula Occludens-1 (ZO-1). These proteins seal the space between epithelial cells and control the selective passage of water, ions, and nutrients, while preventing the uncontrolled translocation of pathogens and inflammatory compounds. Studies have shown that Nano-ZnO consumption increases the strength of the epithelial barrier and reduces paracellular permeability by increasing the expression of genes and tight junction proteins. As a result, the possibility of bacteria, endotoxins, and other inflammatory molecules being transferred from the intestine to the bloodstream is reduced, and local and systemic inflammation is limited (13, 125). The enhancement of epithelial barrier function is not limited to the regulation of tight junction proteins, but also the maintenance and regeneration of epithelial cells plays a crucial role in this process. Increased zinc bioavailability after Nano-ZnO administration facilitates crypt cell proliferation, enterocyte differentiation, and the synthesis of epithelial structural proteins. These changes, together with the reduction of oxidative stress-induced apoptosis, preserve the mucosal structure and accelerate epithelial repair after injury. In many studies, these processes have been associated with increased villus height, decreased crypt depth, and increased villus height to crypt depth ratio; These are known as important markers of mucosal health and nutrient absorption capacity (59, 72, 74, 81, 98, 105, 115, 125). Maintaining the integrity of the intestinal barrier is closely related to the antioxidant capacity of epithelial cells. Oxidative stress is one of the most important factors in the degradation of tight junction proteins and increased mucosal permeability. As explained in the section on Nrf2 pathway activation, Nano-ZnO can prevent oxidative damage to cell membranes and tight junction proteins by increasing the activity of antioxidant enzymes, and consequently the structural stability of the epithelium is maintained and the ability of the mucosa to regenerate under conditions of heat stress, oxidative stress, or microbial challenges is increased (13, 126, 127). In addition, maintaining the function of the epithelial barrier is also directly related to the regulation of inflammatory responses. Excessive activation of the NF-κB pathway and increased production of proinflammatory cytokines such as TNF-*α*, IL-1β, and IL-6 can disrupt the assembly of tight junction proteins and increase intestinal permeability. By inhibiting this pathway and reducing inflammation, Nano-ZnO prevents the destruction of intercellular junctions and provides more favorable conditions for maintaining mucosal integrity. On the other hand, reducing intestinal permeability limits the entry of antigens and endotoxins into the lamina propria, thereby reducing the ongoing stimulation of the immune system. This two-way interaction suggests that maintaining the epithelial barrier and controlling inflammation are highly interdependent processes that act synergistically (13, 79, 97, 106, 125). The health of the epithelial barrier also has a significant impact on the composition and function of the intestinal microbiota. Reducing mucosal permeability and maintaining the epithelial layer create a more stable environment for the establishment of symbiotic bacteria and prevent the colonization of pathogens. In contrast, the increase in beneficial bacteria and their metabolites, especially SCFA, promotes epithelial remodeling and expression of tight junction proteins. Thus, intestinal barrier function, microbiota composition, and mucosal immunity are three interconnected components that reciprocally regulate each other and collectively maintain intestinal homeostasis, a topic discussed in more detail in the section on the interaction of microbiota and host immunity (13, 74, 93–96, 111, 125–130). In recent years, in addition to Nano-ZnO, other new technologies have been developed with the aim of improving intestinal health. Among them, Potentiated Porous ZnO (PP-ZnO) and some organic zinc sources have been able to increase zinc bioavailability without the use of nanotechnology and have shown promising results by enhancing epithelial barrier function, reducing mucosal permeability, modulating immune responses, and reducing zinc excretion into the environment. For example, Marchetti et al. (124) showed that PP-ZnO reproduces many of the protective effects of Nano-ZnO on intestinal health, although the zinc release mechanism and physicochemical properties of the two technologies are different. These findings suggest that the main advantage of Nano-ZnO is not limited to its nanoscale size, but rather its ability to enhance zinc bioavailability and simultaneously regulate multiple molecular pathways (123–126). Overall, the available evidence suggests that the beneficial effects of Nano-ZnO on gut health are not the result of a single mechanism, but rather a coordinated interaction between increased zinc absorption, maintenance of tight junction proteins, enhanced antioxidant capacity, inhibition of inflammation, accelerated epithelial remodeling, and improved interactions between the microbiota and the mucosal immune system. This interconnected network maintains intestinal homeostasis, increases the efficiency of nutrient absorption, and reduces the metabolic cost of inflammation, ultimately explaining a significant portion of the improved growth performance and health of broiler chickens. However, direct comparisons of Nano-ZnO with other novel zinc supplementation technologies are still limited, and studies that simultaneously assess bioavailability, regulation of epithelial barrier-related genes, mucosal safety, economic cost, and environmental implications could help determine the true place of each of these technologies in poultry nutrition (125).

## Summary and future perspective

Despite significant progress in the development of novel zinc supplements, comprehensive comparative data on the efficacy of different technologies under practical conditions in the poultry industry are still lacking. Therefore, one of the most important future research priorities is to conduct comparative studies among advanced zinc delivery technologies, including Nano-ZnO, porous ZnO, potentiated ZnO, organic zinc complexes, encapsulated zinc formulations, and hybrid mineral delivery systems. Such studies should evaluate growth performance, gut health, and immune responses, as well as parameters such as bioavailability, zinc homeostasis, zinc excretion into the environment, long-term safety, tissue accumulation, production cost, and economic efficiency under commercial farming conditions. The results of these studies can lead to the identification of technologies that provide the best balance between bioefficacy, safety, environmental sustainability and economic feasibility and can guide the development of the next generation of mineral supplements in poultry nutrition (13, 123–127).

Furthermore, although the reduction of environmental pollution from zinc excretion is considered one of the most important benefits of modern zinc supplementation technologies, the available comparative data are still limited and heterogeneous. As can be seen in Table 7, most studies have shown that sources with higher bioavailability, especially Nano-ZnO, organic zinc sources and fortified porous zinc oxide, are able to reduce fecal zinc excretion compared to conventional mineral sources by reducing the amount of zinc required in the diet and increasing its retention in the body. However, few studies have directly compared zinc retention, apparent digestibility and fecal excretion under the same conditions. Therefore, standard and long-term commercial-scale studies using quantitative indices of zinc metabolism are essential to accurately assess the environmental benefits of different zinc supplementation technologies (13, 97, 98, 113, 123–127).

Taken together, the available evidence suggests that zinc oxide nanoparticles are one of the most promising zinc supplementation technologies in broiler chicken nutrition due to their high bioavailability and ability to simultaneously regulate multiple molecular pathways, including zinc homeostasis, antioxidant pathways (Nrf2), inflammatory responses (NF-κB), epithelial barrier function, mucosal immunity, and gut microbiota. However, the heterogeneity of study results suggests that biological responses to Nano-ZnO depend on factors such as particle size and physicochemical properties, synthesis method, dosage, diet composition, environmental conditions, and physiological status of the birds (Figure 3). Therefore, determining the optimal dose, assessing long-term safety, investigating the possibility of tissue accumulation, and developing uniform standards for the production and application of these nanoparticles are still among the most important challenges ahead (13, 79, 97, 100, 109, 113, 119, 125).

From a prospective research perspective, moving from descriptive studies to complementary research and studies conducted in real-world commercial farms is essential to more accurately understand the mechanisms of action of Nano-ZnO. Also, conducting comparative studies between new zinc delivery technologies, including zinc oxide nanoparticles, porous zinc oxide, reinforced zinc oxide, organic sources, encapsulated formulations, and hybrid mineral delivery systems, can lead to the identification of solutions that create the best balance between biological efficacy, safety, environmental sustainability, and economic justification. Finally, the integration of new zinc supplementation technologies with precision feeding strategies and smart flock management can play an important role in developing sustainable poultry production systems, reducing mineral resource consumption, reducing environmental pollution, and improving the health and productivity of the poultry industry in the future (13, 123–135).

## Conclusion

Nanotechnology represents a promising approach to improve the efficiency of trace mineral supplementation in poultry nutrition. Among the various nano-based feed additives, nano zinc has attracted considerable attention because of its potential to enhance zinc bioavailability while reducing dietary inclusion levels and environmental zinc excretion. Current evidence suggests that nano zinc supplementation may positively influence growth performance, antioxidant status, immune function, intestinal health, and selected blood biochemical parameters under specific experimental conditions. These beneficial effects are generally attributed to improved zinc absorption and utilization, although the underlying molecular mechanisms remain incompletely understood. In addition to its nutritional benefits, improved zinc utilization may contribute to more sustainable poultry production by decreasing mineral excretion and the associated environmental burden. Furthermore, the antimicrobial and immunomodulatory properties of nano zinc suggest potential applications within integrated strategies to improve intestinal health and reduce reliance on antimicrobial compounds. However, the currently available evidence does not yet support nano zinc as a direct replacement for antibiotics in commercial poultry production. Despite these encouraging findings, several important knowledge gaps remain. Future research should focus on defining the optimal particle characteristics and dietary inclusion levels, elucidating the molecular mechanisms underlying the observed biological responses, evaluating long-term safety and tissue accumulation, and establishing standardized recommendations for practical application under commercial production conditions. Addressing these questions will be essential before nano zinc can be widely implemented as a sustainable nutritional strategy in broiler production.
