# Correction: Nano zinc supplementation in broiler nutrition: current evidence, mechanisms of action, and future perspectives

**DOI:** 10.3389/fvets.2026.2017168

**Published:** 2026-10-13

**Authors:** Frontiers Production Office

**Affiliations:** Frontiers Media SA, Lausanne, Switzerland

**Keywords:** bioavailability, broiler chickens, immune function, intestinal health, oxidative stress, sustainability, zinc nanoparticles

The funder State Scientific Institution NIIMMP, grant RSF No. 22-16-00041-P was erroneously omitted.

The original version of this article has been updated.

